# Long-lasting infection with *Anaplasma ovis* in sheep

**DOI:** 10.1007/s11259-023-10186-y

**Published:** 2023-08-02

**Authors:** Héctor Ruiz, Marta Ruiz de Arcaute, Alfredo Ángel Benito, Sergio Villanueva-Saz, José Calasanz Jiménez, Delia Lacasta

**Affiliations:** 1 Animal Pathology Department, Instituto Agroalimentario de Aragón-IA2, Universidad de Zaragoza-CITA, C. Miguel Servet 177, Zaragoza, 50013 Spain; 2 Veterinary Faculty, University of Zaragoza, Zaragoza, 50013 Spain; 3 Exopol S.L, Pol. Río Gállego D-14, Zaragoza, 50840 Spain; 4 Veterinarios Asesores en Clínica y Sanidad Animal S.L, C. Ramón y Cajal 14, Zaragoza, 50003 Spain

**Keywords:** *Anaplasma ovis*, Long-term infection, Ovine anaplasmosis, PCR, Sheep, Tick-borne disease

## Abstract

Ovine anaplasmosis is an emerging vector-borne disease in Europe caused by *Anaplasma ovis*. The infection has spread quickly in recent years, causing moderate to severe outbreaks in sheep flocks, leading to relevant economic losses in sheep farming. This wider spread has been associated with global warming and climate change, favouring the maintenance and life cycle of their main vector, the ticks. However, another epidemiological aspect could favour this quick spread. Long persistence infection of *Anaplasma ovis* has been proposed as a hypothesis in several articles but never scientifically proven. The results of the present study demonstrate that eight adult sheep, both naturally or experimentally infected, maintain *Anaplasma ovis* load in blood during their whole productive life (4 to 6 years), being permanently infected. In addition, the results suggest that *A. ovis* bacterial load can be constant or suffer fluctuations, as has been demonstrated in other *Anaplasma* species. Both aspects can be determinants in the epidemiology and the transmission of the infection.

**Supplementary Information:**

The online version contains supplementary material available at 10.1007/s11259-023-10186-y.

## Introduction

Ovine anaplasmosis is an emerging disease in Europe, caused by the bacteria *Anaplasma ovis* and mainly transmitted by ticks. The disease has been recently reported in some European countries as Bulgaria, France, Greece, Italy, Romania, Spain and almost all of the Mediterranean Basin (Stuen 2016).

*Anaplasma* is a genus belonging to the order Rickettsiales which causes vector-borne diseases in mammals (Rar and Golovljova 2011). Specifically, *Anaplasma ovis* is an obligate intraerythrocytic, Gram-negative bacteria that has been diagnosed in small ruminants, both domestic and wild, although it is considered a potential zoonotic pathogen (Chochlakis et al. 2010).

Ovine anaplasmosis is characterised by unspecific clinical signs such as weakness, anorexia, weight loss, lower milk production, anaemia, pallor of mucous membranes and slight fever peaks. Other clinical signs, such as epiphora and haematuria, can also be observed in severely affected flocks (Lacasta et al. 2021; Yasini et al. 2012). However, sometimes *A. ovis* is silently transmitted without causing apparent clinical signs.

Initially, the examination of whole blood smears was the most used diagnostic test, where basophilic bodies were observed in light microscopes inside the erythrocytes (Eriks et al. 1989). However, this technique is obsolete. Currently, the most common diagnostic test is the polymerase chain reaction (PCR), a molecular test capable of detecting the *msp4* gene, allowing to differentiate *A. ovis* from other *Anaplasma* species (De la Fuente et al. 2002; Torina et al. 2012). Serological techniques are limited to competitive Enzyme-Linked Immunosorbent Assay (ELISA) methods. However, these tests are based on Msp5 protein, being not able to differentiate between *Anaplasma* species since this protein is common in all species (Mason et al. 2017; Lacasta et al. 2021).

Mechanical transmission by tabanids, fleas and even needles is considered possible (Mason et al. 2017). However, it is clear that Ixodidae family ticks are the most crucial vector involved in the transmission, being considered the biological vector of these diseases because the bacteria are able to replicate in the salivary glands of the tick, enhancing the infective potential (Kocan et al. 2004).

The disease has quickly spread in Europe, as has been demonstrated due to the high number of recent outbreaks reported (Stuen 2016). Some factors have been associated with this spread. Global warming, climate change and the drier and warmer weather conditions have favoured the life cycle of ticks, being alive for almost the whole year in some areas of the Mediterranean Basin. In addition, the increase in the number of wildlife ruminants that can act as reservoirs of infection may also be another essential factor. Even more, if there is close contact in habitats with livestock. The migratory animals can also participate in this spread, carrying affected vectors to naïve areas (Bouchard et al. 2019).

It has been suggested by some authors that long bacteremia in animals infected by *A. ovis*. However, permanent infections have only been demonstrated for 2 or 3 years, both in goats and sheep (Palmer et al. 1998; Jiménez et al. 2019). The present work intends to analyse whether animals infected by *A. ovis* are really permanently infected if proper antibiotic treatment is not applied. In order to demonstrate this hypothesis, 11 ewes were analysed during their whole productive life (between 4 and 6 years of life).

## Materials and methods

### Animals

Eight out of eleven of the analysed animals were *Anaplasma ovis* positive at the beginning of the experiment, either by experimental or natural infection. Three of these animals (126, 127, 128) had been *A. ovis* experimentally infected in 2015, after Spain’s first ovine anaplasmosis outbreak, and two of their female offspring (542, 543) were experimentally infected two years later (Jiménez et al. 2019). All experimentally infected ewes were intravenously inoculated with a single dose of 30ml of whole blood mixed of two naturally infected ewes between 8.35 × 10^6^ and 1.18 × 10^7^* A. ovis* copies per mililiter. Simultaneously, three positive animals belonging to naturally infected herds close to our facilities in Zaragoza were included in the present study in the years 2015 (175) and 2016 (341, 355) to follow up on the development of the natural infection. In addition, three clinically healthy and *A. ovis* negative ewes of the faculty herd were included as a control group. All the animals were kept indoors throughout the experiment, without access to pasture, living with other teaching flock animals in the Zaragoza Veterinary Faculty facilities.

### Clinical exam and sample collection

Clinical follow-up was performed monthly on all animals throughout the duration of the study (between 4 and 6 years), and blood samples with anticoagulant (EDTA) were collected every six months from the jugular vein through a vacutainer system and frozen at -30ºC for further analysis. In addition, a physical examination was performed on each animal to detect ticks or/and other biological vectors at the sampling moment.

### Molecular tests

The commercial kit, MagMAX™ Pathogen RNA/DNA (Thermo Fisher Scientific, Austin, TX, USA) with an automated magnetic particle processor (KingFisher Flex System, Thermo Fisher Scientific, Vantaa, Finland), was used for nucleic acids extraction according to the manufacturer’s instructions. Amplification was carried out in a 7500 fast real-time PCR machine (Applied Biosystems, Marsiling, Singapore), and results were analysed with the respective software (7500 software v2.3, Foster, CA, USA). The specific detection of *A. ovis* was carried out by using the commercial kit EXOone Anaplasma ovis (EXOPOL S.L., San Mateo de Gállego, Spain) that targets the single copy *msp4* gene and following the manufacturer’s instructions. This qPCR assay has an analytical sensitivity of 50 copies of genomic equivalent/reaction and includes a quantified synthetic positive control. Additionally, an endogenous control was also included in all of the assays in order to avoid false-negative results. The bacterial load was expressed using the quantification cycle (Cq), which is the cycle number where the PCR amplification curve intersects the threshold line (Bustin et al. 2009). A relative quantification was performed based on the Cq value and the quantified positive control (5,00E + 05eg/rxn) provided with the kit, using the mathematical equation $\text{X}=2\text{*}10^14\text{*}2.7182^(-0.682\text{*}\text{C}\text{q} \text{v}\text{a}\text{l}\text{u}\text{e}).$

## Results

All animals that started the study being *A. ovis* positive continued to be positive until the end of the experiment, and all the negative animals were negative throughout the analysed period (Table 1). No ticks or other possible vectors less than fleas or flies were observed during the trial, either in the animals or in the facilities. No clinical signs associated with ovine anaplasmosis were observed in the animals during the study.

**Table 1:** Evolution of Cq values in qPCR *Anaplasma ovis* test performed periodically every 6 months. Values lower than 38 are considered positive. † means dead during the study

ID/DATE	2015/2	2016/1	2016/2	2017/1	2017/2	2018/1	2018/2	2019/1	2019/2	2020/1	2020/2	2021/1	2021/2
126	26	27	27	28	30	28	32	29	29	26	27	29	28
127	26	25	26	30	31	28	31	37	30	29	35	27	28
128	29	27	25	28	31	30	35	25	29	33	29	38	31
175	27	25	26	25	27	27	30	25	27	29	26	27	29
341	-	-	29	25	30	34	31	30	31	29	27	29	25
355	-	-	30	25	30	24	31	32	27	28	28	30	27
542	-	-	-	27	28	27	27	26	27	28	29	29	28
543	-	-	-	28	29	29	29	27	28	29	27	28	27
358	-	-	NEG	NEG	NEG	NEG	NEG	NEG	NEG	NEG	NEG	NEG	**†**
57,148	-	-	NEG	NEG	NEG	NEG	NEG	NEG	NEG	NEG	NEG	NEG	NEG
289	-	-	-	-	NEG	NEG	NEG	NEG	NEG	NEG	NEG	NEG	NEG

**Fig. 1:**
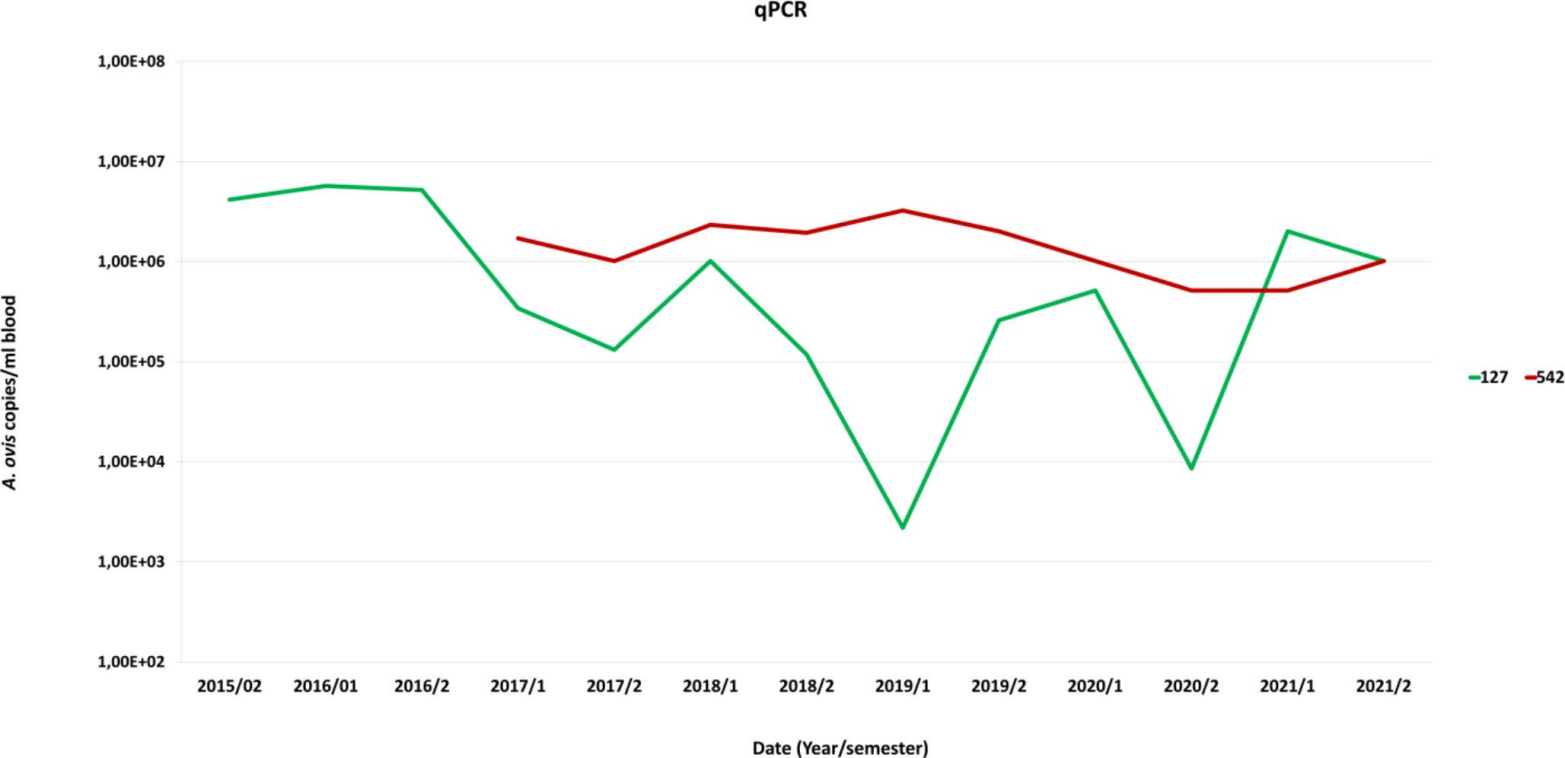
An Example of qPCR results show a fluctuating *A. ovis* load in animal 127 and a constant a constant *A. ovis* load near to 1,00E + 06 during the whole life of the animal 543

Different degrees of fluctuation were observed, both in naturally and experimentally infected animals, showing the development of the bacterial load throughout the analysed period. Three of the animals (175, 542 and 543) presented a constant bacterial load throughout all their lives, and the rest of the positive animals showed fluctuations in the bacterial load throughout the studied period (Fig. 1).

## Discussion

Our results confirm that positive animals remain carriers for at least six years, confirming the initial hypothesis. Previous studies had shown a permanent infection for 2.5 years (Jiménez et al. 2019; Yasini et al. 2012). However, the present study demonstrates a permanent infection throughout the animal’s productive life. This is a crucial aspect in the spread of ovine anaplasmosis. If all the animals that come into contact with the bacteria remain permanently infected, they become an important source for the spread of the infection since every time a tick ingests blood from these animals, it could become a new vector of the disease, making it even easier to transmit due to the transstadial transmission of *Anaplasma* species affecting sheep in ticks (Taank et al. 2020).

Furthermore, if the sheep remain carriers for life, they favour both the endemicity of the disease in the affected herd and the spread of the disease (Jiménez et al. 2019). That hypothesis could be consistently supported by both factors: a passive immunity provided by colostrum and the high percentage of infected animals favouring enzootic stability in herds (Corona et al. 2005; Lacasta et al. 2021). Only after some stressful factors as heat stress, lack of available food, etc., can this endemicity be interrupted (Renneker et al. 2013).

The bacterial load fluctuations through their lives could be associated with climatic changes, productive status (parturition, pregnancy, lactation), stressful moments, coinfections, etc. Accordingly to these results, in other species, undulating bacteremia periods have been described. *A. marginale*-infected cattle and *A. ovis-*infected goats may suffer bacteremia cycles with a maximum peak every 6 to 8 weeks (Eid et al. 1996; Wang et al. 2017). However, more studies based on the bacteriemia cycles observed in other *Anaplasm*a species should be done in *A. ovis* infections to confirm it. Even more, these researchs should also take into account the possible stressful factor effects in the bacteriemia cycles and possible reactivation of clinical signs in long-term infected animals.

In conclusion, our results indicated that animals affected by ovine anaplasmosis can be permanently infected without demonstrating clinical signs for up to 6 years. In natural conditions, these animals can act as reservoirs of the disease, favouring the spread of the infection. However, this aspect can help to reach endemicity in the flock due to the natural immunity and the high intraherd-prevalence levels.

### Electronic supplementary material

Below is the link to the electronic supplementary material.

Supplementary Material 1

## Data Availability

The data that support the findings of this study are available from the corresponding author upon reasonable request.
