# Supplementary figures and images for: Long-lasting infection with Anaplasma ovis in sheep
Source: Vet Res Commun. 2023 Aug 2;48(1):521–5. doi: 10.1007/s11259-023-10186-y (PMC10810980; doi:10.1007/s11259-023-10186-y)

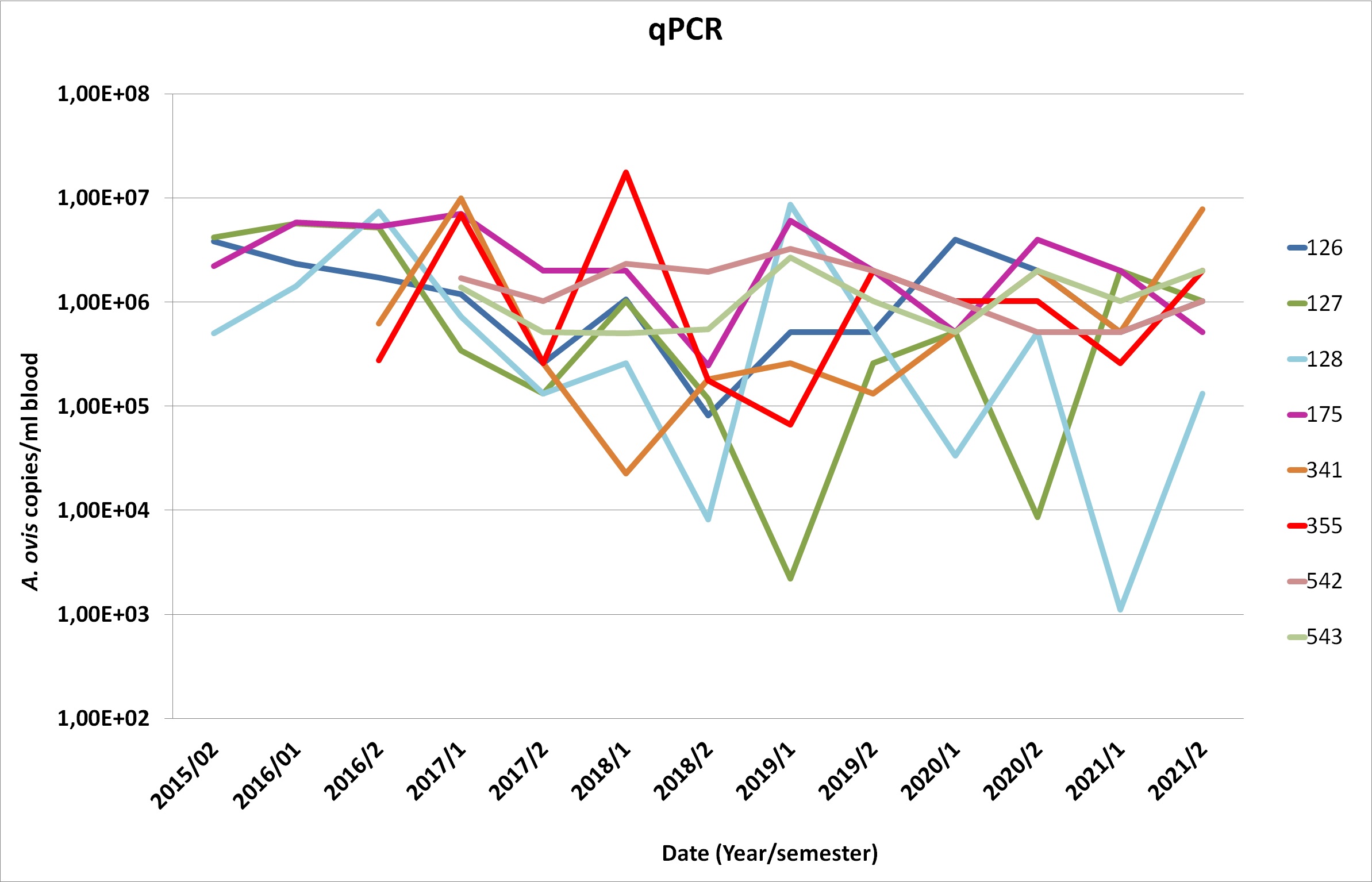

Supplement: Supplementary file 1 — Supplementary Material 1 [file 11259_2023_10186_MOESM1_ESM.jpg]
